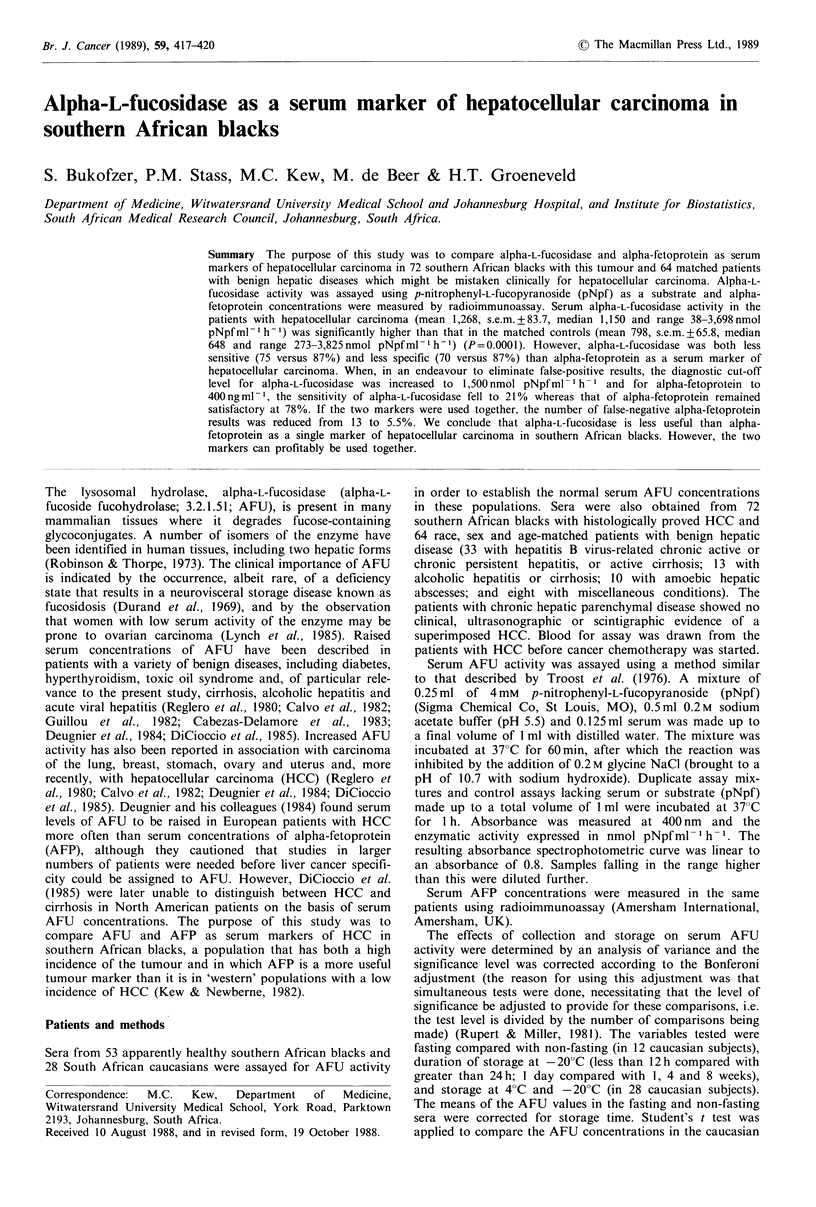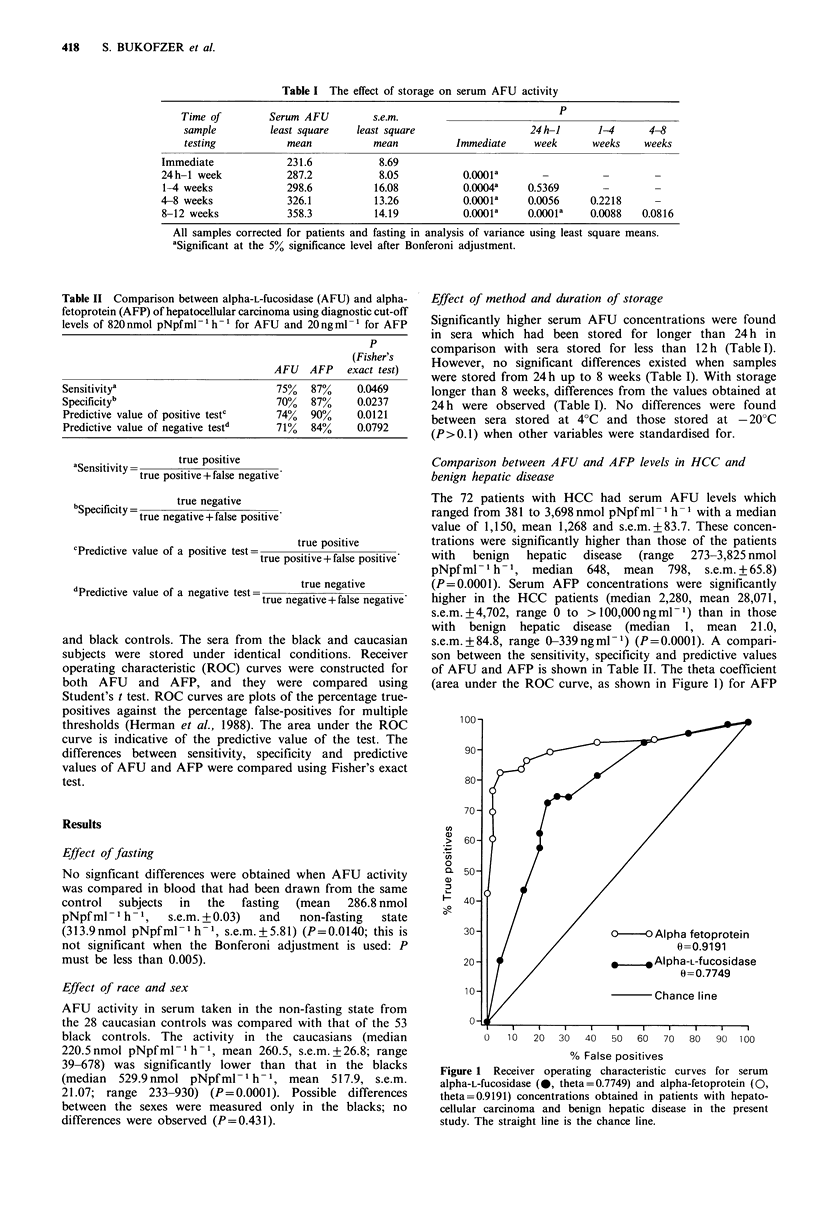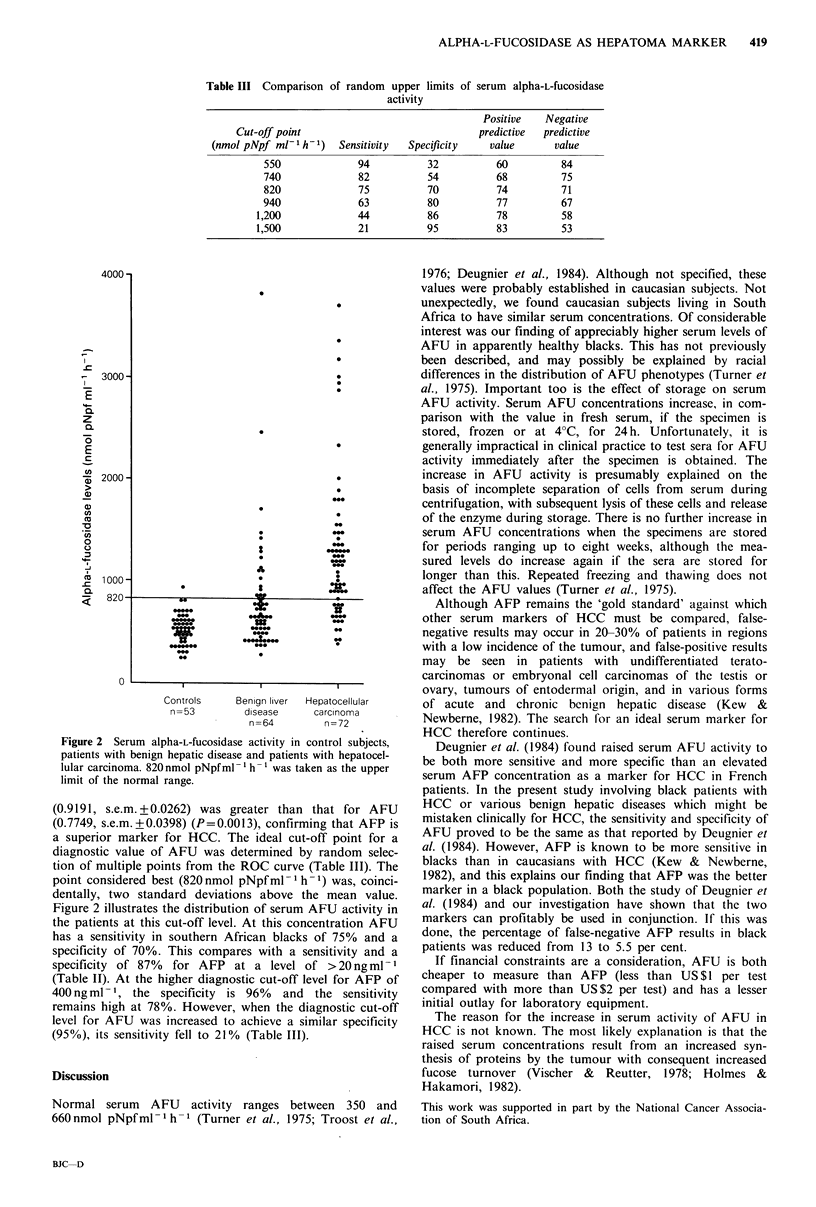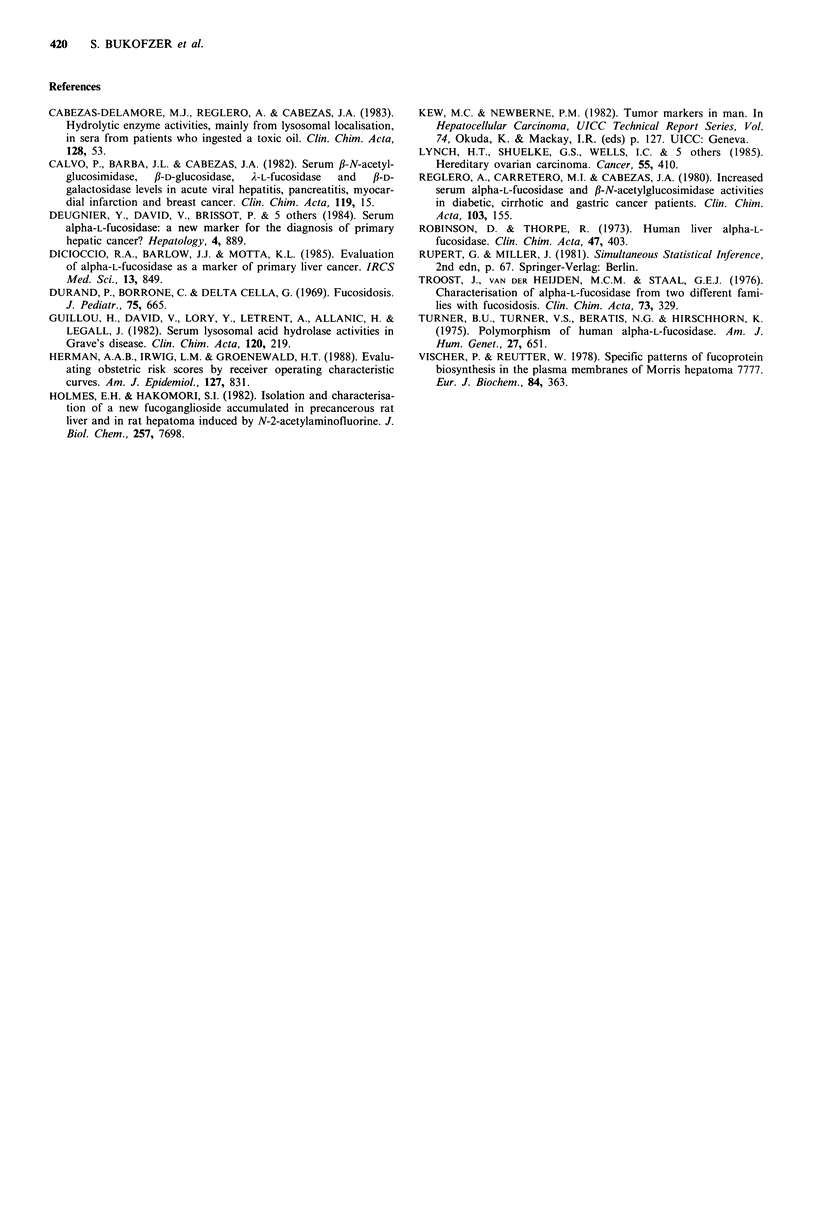# Alpha-L-fucosidase as a serum marker of hepatocellular carcinoma in southern African blacks.

**DOI:** 10.1038/bjc.1989.84

**Published:** 1989-03

**Authors:** S. Bukofzer, P. M. Stass, M. C. Kew, M. de Beer, H. T. Groeneveld

**Affiliations:** Department of Medicine, Witwatersrand University Medical School, Johannesburg, South Africa.

## Abstract

The purpose of this study was to compare alpha-L-fucosidase and alpha-fetoprotein as serum markers of hepatocellular carcinoma in 72 southern African blacks with this tumour and 64 matched patients with benign hepatic diseases which might be mistaken clinically for hepatocellular carcinoma. Alpha-L-fucosidase activity was assayed using p-nitrophenyl-L-fucopyranoside (pNpf) as a substrate and alpha-fetoprotein concentrations were measured by radioimmunoassay. Serum alpha-L-fucosidase activity in the patients with hepatocellular carcinoma (mean 1,268, s.e.m. +/- 83.7, median 1,150 and range 38-3,698 nmol pNpf ml-1 h-1) was significantly higher than that in the matched controls (mean 798, s.e.m. +/- 65.8, median 648 and range 273-3,825 nmol pNpf ml-1 h-1) (P = 0.0001). However, alpha-L-fucosidase was both less sensitive (75 versus 87%) and less specific (70 versus 87%) than alpha-fetoprotein as a serum marker of hepatocellular carcinoma. When, in an endeavour to eliminate false-positive results, the diagnostic cut-off level for alpha-L-fucosidase was increased to 1,500 nmol pNpf ml-1 h-1 and for alpha-fetoprotein to 400 ng ml-1, the sensitivity of alpha-L-fucosidase fell to 21% whereas that of alpha-fetoprotein remained satisfactory at 78%. If the two markers were used together, the number of false-negative alpha-fetoprotein results was reduced from 13 to 5.5%. We conclude that alpha-L-fucosidase is less useful than alpha-fetoprotein as a single marker of hepatocellular carcinoma in southern African blacks. However, the two markers can profitably be used together.


Br. J. Cancer (1989), 59, 417–420 © The Macmillan Press Ltd., 1989

### Alpha-L-fucosidase as a serum marker of hepatocellular carcinoma in southern African blacks

S. Bukofzer, P.M. Stass, M.C. Kew, M. de Beer & H.T. Groeneveld

*Department of Medicine, Witwatersrand University Medical School and Johannesburg Hospital, and Institute for Biostatistics, South African Medical Research Council, Johannesburg, South Africa.*

**Summary** The purpose of this study was to compare alpha-L-fucosidase and alpha-fetoprotein as serum markers of hepatocellular carcinoma in 72 southern African blacks with this tumour and 64 matched patients with benign hepatic diseases which might be mistaken clinically for hepatocellular carcinoma. Alpha-L-fucosidase activity was assayed using *p*-nitrophenyl-L-fucopyranoside (pNpf) as a substrate and alpha-fetoprotein concentrations were measured by radioimmunoassay. Serum alpha-L-fucosidase activity in the patients with hepatocellular carcinoma (mean 1,268, s.e.m. $\pm$83.7, median 1,150 and range 38–3,698 nmol pNpf $ml^{-1} h^{-1}$) was significantly higher than that in the matched controls (mean 798, s.e.m. $\pm$65.8, median 648 and range 273–3,825 nmol pNpf $ml^{-1} h^{-1}$) ($P$=0.0001). However, alpha-L-fucosidase was both less sensitive (75 versus 87%) and less specific (70 versus 87%) than alpha-fetoprotein as a serum marker of hepatocellular carcinoma. When, in an endeavour to eliminate false-positive results, the diagnostic cut-off level for alpha-L-fucosidase was increased to 1,500 nmol pNpf $ml^{-1} h^{-1}$ and for alpha-fetoprotein to 400 ng $ml^{-1}$, the sensitivity of alpha-L-fucosidase fell to 21% whereas that of alpha-fetoprotein remained satisfactory at 78%. If the two markers were used together, the number of false-negative alpha-fetoprotein results was reduced from 13 to 5.5%. We conclude that alpha-L-fucosidase is less useful than alpha-fetoprotein as a single marker of hepatocellular carcinoma in southern African blacks. However, the two markers can profitably be used together.

The lysosomal hydrolase, alpha-L-fucosidase (alpha-L-fucoside fucohydrolase; 3.2.1.51; AFU), is present in many mammalian tissues where it degrades fucose-containing glycoconjugates. A number of isomers of the enzyme have been identified in human tissues, including two hepatic forms (Robinson & Thorpe, 1973). The clinical importance of AFU is indicated by the occurrence, albeit rare, of a deficiency state that results in a neurovisceral storage disease known as fucosidosis (Durand *et al.*, 1969), and by the observation that women with low serum activity of the enzyme may be prone to ovarian carcinoma (Lynch *et al.*, 1985). Raised serum concentrations of AFU have been described in patients with a variety of benign diseases, including diabetes, hyperthyroidism, toxic oil syndrome and, of particular relevance to the present study, cirrhosis, alcoholic hepatitis and acute viral hepatitis (Reglero *et al.*, 1980; Calvo *et al.*, 1982; Guillou *et al.*, 1982; Cabezas-Delamore *et al.*, 1983; Deugnier *et al.*, 1984; DiCioccio *et al.*, 1985). Increased AFU activity has also been reported in association with carcinoma of the lung, breast, stomach, ovary and uterus and, more recently, with hepatocellular carcinoma (HCC) (Reglero *et al.*, 1980; Calvo *et al.*, 1982; Deugnier *et al.*, 1984; DiCioccio *et al.*, 1985). Deugnier and his colleagues (1984) found serum levels of AFU to be raised in European patients with HCC more often than serum concentrations of alpha-fetoprotein (AFP), although they cautioned that studies in larger numbers of patients were needed before liver cancer specificity could be assigned to AFU. However, DiCioccio *et al.* (1985) were later unable to distinguish between HCC and cirrhosis in North American patients on the basis of serum AFU concentrations. The purpose of this study was to compare AFU and AFP as serum markers of HCC in southern African blacks, a population that has both a high incidence of the tumour and in which AFP is a more useful tumour marker than it is in 'western' populations with a low incidence of HCC (Kew & Newberne, 1982).

### Patients and methods

Sera from 53 apparently healthy southern African blacks and 28 South African caucasians were assayed for AFU activity in order to establish the normal serum AFU concentrations in these populations. Sera were also obtained from 72 southern African blacks with histologically proved HCC and 64 race, sex and age-matched patients with benign hepatic disease (33 with hepatitis B virus-related chronic active or chronic persistent hepatitis, or active cirrhosis; 13 with alcoholic hepatitis or cirrhosis; 10 with amoebic hepatic abscesses; and eight with miscellaneous conditions). The patients with chronic hepatic parenchymal disease showed no clinical, ultrasonographic or scintigraphic evidence of a superimposed HCC. Blood for assay was drawn from the patients with HCC before cancer chemotherapy was started.

Serum AFU activity was assayed using a method similar to that described by Troost *et al.* (1976). A mixture of 0.25 ml of 4 mM *p*-nitrophenyl-L-fucopyranoside (pNpf) (Sigma Chemical Co, St Louis, MO), 0.5 ml 0.2 M sodium acetate buffer (pH 5.5) and 0.125 ml serum was made up to a final volume of 1 ml with distilled water. The mixture was incubated at 37°C for 60 min, after which the reaction was inhibited by the addition of 0.2 M glycine NaCl (brought to a pH of 10.7 with sodium hydroxide). Duplicate assay mixtures and control assays lacking serum or substrate (pNpf) made up to a total volume of 1 ml were incubated at 37°C for 1 h. Absorbance was measured at 400 nm and the enzymatic activity expressed in nmol pNpf $ml^{-1} h^{-1}$. The resulting absorbance spectrophotometric curve was linear to an absorbance of 0.8. Samples falling in the range higher than this were diluted further.

Serum AFP concentrations were measured in the same patients using radioimmunoassay (Amersham International, Amersham, UK).

The effects of collection and storage on serum AFU activity were determined by an analysis of variance and the significance level was corrected according to the Bonferoni adjustment (the reason for using this adjustment was that simultaneous tests were done, necessitating that the level of significance be adjusted to provide for these comparisons, i.e. the test level is divided by the number of comparisons being made) (Rupert & Miller, 1981). The variables tested were fasting compared with non-fasting (in 12 caucasian subjects), duration of storage at −20°C (less than 12 h compared with greater than 24 h; 1 day compared with 1, 4 and 8 weeks), and storage at 4°C and −20°C (in 28 caucasian subjects). The means of the AFU values in the fasting and non-fasting sera were corrected for storage time. Student's *t* test was applied to compare the AFU concentrations in the caucasian

Correspondence: M.C. Kew, Department of Medicine, Witwatersrand University Medical School, York Road, Parktown 2193, Johannesburg, South Africa.
Received 10 August 1988, and in revised form, 19 October 1988.

**Table I** The effect of storage on serum AFU activity

| *Time of sample testing* | *Serum AFU least square mean* | *s.e.m. least square mean* | *P* Immediate | *24 h–1 week* | *1–4 weeks* | *4–8 weeks* |
|---|---|---|---|---|---|---|
| Immediate | 231.6 | 8.69 | | | | |
| 24 h–1 week | 287.2 | 8.05 | 0.0001[a] | – | – | – |
| 1–4 weeks | 298.6 | 16.08 | 0.0004[a] | 0.5369 | – | – |
| 4–8 weeks | 326.1 | 13.26 | 0.0001[a] | 0.0056 | 0.2218 | – |
| 8–12 weeks | 358.3 | 14.19 | 0.0001[a] | 0.0001[a] | 0.0088 | 0.0816 |

All samples corrected for patients and fasting in analysis of variance using least square means. [a]Significant at the 5% significance level after Bonferoni adjustment.

**Table II** Comparison between alpha-L-fucosidase (AFU) and alpha-fetoprotein (AFP) of hepatocellular carcinoma using diagnostic cut-off levels of 820 nmol pNpf ml$^{-1}$ h$^{-1}$ for AFU and 20 ng ml$^{-1}$ for AFP

| | *AFU* | *AFP* | *P (Fisher's exact test)* |
|---|---|---|---|
| Sensitivity[a] | 75% | 87% | 0.0469 |
| Specificity[b] | 70% | 87% | 0.0237 |
| Predictive value of positive test[c] | 74% | 90% | 0.0121 |
| Predictive value of negative test[d] | 71% | 84% | 0.0792 |

[a]Sensitivity $= \frac{\text{true positive}}{\text{true positive} + \text{false negative}}$.

[b]Specificity $= \frac{\text{true negative}}{\text{true negative} + \text{false positive}}$.

[c]Predictive value of a positive test $= \frac{\text{true positive}}{\text{true positive} + \text{false positive}}$.

[d]Predictive value of a negative test $= \frac{\text{true negative}}{\text{true negative} + \text{false negative}}$.

and black controls. The sera from the black and caucasian subjects were stored under identical conditions. Receiver operating characteristic (ROC) curves were constructed for both AFU and AFP, and they were compared using Student's *t* test. ROC curves are plots of the percentage true-positives against the percentage false-positives for multiple thresholds (Herman *et al.*, 1988). The area under the ROC curve is indicative of the predictive value of the test. The differences between sensitivity, specificity and predictive values of AFU and AFP were compared using Fisher's exact test.

### Results

### *Effect of fasting*

No signficant differences were obtained when AFU activity was compared in blood that had been drawn from the same control subjects in the fasting (mean 286.8 nmol pNpf ml$^{-1}$ h$^{-1}$, s.e.m. $\pm$0.03) and non-fasting state (313.9 nmol pNpf ml$^{-1}$ h$^{-1}$, s.e.m. $\pm$5.81) ($P = 0.0140$; this is not significant when the Bonferoni adjustment is used: $P$ must be less than 0.005).

### *Effect of race and sex*

AFU activity in serum taken in the non-fasting state from the 28 caucasian controls was compared with that of the 53 black controls. The activity in the caucasians (median 220.5 nmol pNpf ml$^{-1}$ h$^{-1}$, mean 260.5, s.e.m. $\pm$26.8; range 39–678) was significantly lower than that in the blacks (median 529.9 nmol pNpf ml$^{-1}$ h$^{-1}$, mean 517.9, s.e.m. 21.07; range 233–930) ($P = 0.0001$). Possible differences between the sexes were measured only in the blacks; no differences were observed ($P = 0.431$).

### *Effect of method and duration of storage*

Significantly higher serum AFU concentrations were found in sera which had been stored for longer than 24 h in comparison with sera stored for less than 12 h (Table I). However, no significant differences existed when samples were stored from 24 h up to 8 weeks (Table I). With storage longer than 8 weeks, differences from the values obtained at 24 h were observed (Table I). No differences were found between sera stored at 4°C and those stored at −20°C ($P > 0.1$) when other variables were standardised for.

### *Comparison between AFU and AFP levels in HCC and benign hepatic disease*

The 72 patients with HCC had serum AFU levels which ranged from 381 to 3,698 nmol pNpf ml$^{-1}$ h$^{-1}$ with a median value of 1,150, mean 1,268 and s.e.m. $\pm$83.7. These concentrations were significantly higher than those of the patients with benign hepatic disease (range 273–3,825 nmol pNpf ml$^{-1}$ h$^{-1}$, median 648, mean 798, s.e.m. $\pm$65.8) ($P = 0.0001$). Serum AFP concentrations were significantly higher in the HCC patients (median 2,280, mean 28,071, s.e.m. $\pm$4,702, range 0 to >100,000 ng ml$^{-1}$) than in those with benign hepatic disease (median 1, mean 21.0, s.e.m. $\pm$84.8, range 0–339 ng ml$^{-1}$) ($P = 0.0001$). A comparison between the sensitivity, specificity and predictive values of AFU and AFP is shown in Table II. The theta coefficient (area under the ROC curve, as shown in Figure 1) for AFP

**Figure 1** Receiver operating characteristic curves for serum alpha-L-fucosidase (●, theta = 0.7749) and alpha-fetoprotein (○, theta = 0.9191) concentrations obtained in patients with hepatocellular carcinoma and benign hepatic disease in the present study. The straight line is the chance line.

**Table III** Comparison of random upper limits of serum alpha-L-fucosidase activity

| *Cut-off point (nmol pNpf ml$^{-1}$ h$^{-1}$)* | *Sensitivity* | *Specificity* | *Positive predictive value* | *Negative predictive value* |
|---|---|---|---|---|
| 550 | 94 | 32 | 60 | 84 |
| 740 | 82 | 54 | 68 | 75 |
| 820 | 75 | 70 | 74 | 71 |
| 940 | 63 | 80 | 77 | 67 |
| 1,200 | 44 | 86 | 78 | 58 |
| 1,500 | 21 | 95 | 83 | 53 |

**Figure 2** Serum alpha-L-fucosidase activity in control subjects, patients with benign hepatic disease and patients with hepatocellular carcinoma. 820 nmol pNpf ml$^{-1}$ h$^{-1}$ was taken as the upper limit of the normal range.

(0.9191, s.e.m. ±0.0262) was greater than that for AFU (0.7749, s.e.m. ±0.0398) ($P=0.0013$), confirming that AFP is a superior marker for HCC. The ideal cut-off point for a diagnostic value of AFU was determined by random selection of multiple points from the ROC curve (Table III). The point considered best (820 nmol pNpf ml$^{-1}$ h$^{-1}$) was, coincidentally, two standard deviations above the mean value. Figure 2 illustrates the distribution of serum AFU activity in the patients at this cut-off level. At this concentration AFU has a sensitivity in southern African blacks of 75% and a specificity of 70%. This compares with a sensitivity and a specificity of 87% for AFP at a level of >20 ng ml$^{-1}$ (Table II). At the higher diagnostic cut-off level for AFP of 400 ng ml$^{-1}$, the specificity is 96% and the sensitivity remains high at 78%. However, when the diagnostic cut-off level for AFU was increased to achieve a similar specificity (95%), its sensitivity fell to 21% (Table III).

### Discussion

Normal serum AFU activity ranges between 350 and 660 nmol pNpf ml$^{-1}$ h$^{-1}$ (Turner *et al.*, 1975; Troost *et al.*, 1976; Deugnier *et al.*, 1984). Although not specified, these values were probably established in caucasian subjects. Not unexpectedly, we found caucasian subjects living in South Africa to have similar serum concentrations. Of considerable interest was our finding of appreciably higher serum levels of AFU in apparently healthy blacks. This has not previously been described, and may possibly be explained by racial differences in the distribution of AFU phenotypes (Turner *et al.*, 1975). Important too is the effect of storage on serum AFU activity. Serum AFU concentrations increase, in comparison with the value in fresh serum, if the specimen is stored, frozen or at 4°C, for 24 h. Unfortunately, it is generally impractical in clinical practice to test sera for AFU activity immediately after the specimen is obtained. The increase in AFU activity is presumably explained on the basis of incomplete separation of cells from serum during centrifugation, with subsequent lysis of these cells and release of the enzyme during storage. There is no further increase in serum AFU concentrations when the specimens are stored for periods ranging up to eight weeks, although the measured levels do increase again if the sera are stored for longer than this. Repeated freezing and thawing does not affect the AFU values (Turner *et al.*, 1975).

Although AFP remains the 'gold standard' against which other serum markers of HCC must be compared, false-negative results may occur in 20–30% of patients in regions with a low incidence of the tumour, and false-positive results may be seen in patients with undifferentiated teratocarcinomas or embryonal cell carcinomas of the testis or ovary, tumours of entodermal origin, and in various forms of acute and chronic benign hepatic disease (Kew & Newberne, 1982). The search for an ideal serum marker for HCC therefore continues.

Deugnier *et al.* (1984) found raised serum AFU activity to be both more sensitive and more specific than an elevated serum AFP concentration as a marker for HCC in French patients. In the present study involving black patients with HCC or various benign hepatic diseases which might be mistaken clinically for HCC, the sensitivity and specificity of AFU proved to be the same as that reported by Deugnier *et al.* (1984). However, AFP is known to be more sensitive in blacks than in caucasians with HCC (Kew & Newberne, 1982), and this explains our finding that AFP was the better marker in a black population. Both the study of Deugnier *et al.* (1984) and our investigation have shown that the two markers can profitably be used in conjunction. If this was done, the percentage of false-negative AFP results in black patients was reduced from 13 to 5.5 per cent.

If financial constraints are a consideration, AFU is both cheaper to measure than AFP (less than US$1 per test compared with more than US$2 per test) and has a lesser initial outlay for laboratory equipment.

The reason for the increase in serum activity of AFU in HCC is not known. The most likely explanation is that the raised serum concentrations result from an increased synthesis of proteins by the tumour with consequent increased fucose turnover (Vischer & Reutter, 1978; Holmes & Hakamori, 1982).

This work was supported in part by the National Cancer Association of South Africa.

### References

CABEZAS-DELAMORE, M.J., REGLERO, A. & CABEZAS, J.A. (1983). Hydrolytic enzyme activities, mainly from lysosomal localisation, in sera from patients who ingested a toxic oil. *Clin. Chim. Acta*, **128**, 53.

CALVO, P., BARBA, J.L. & CABEZAS, J.A. (1982). Serum $\beta$-*N*-acetylglucosimidase, $\beta$-D-glucosidase, $\lambda$-L-fucosidase and $\beta$-D-galactosidase levels in acute viral hepatitis, pancreatitis, myocardial infarction and breast cancer. *Clin. Chim. Acta*, **119**, 15.

DEUGNIER, Y., DAVID, V., BRISSOT, P. & 5 others (1984). Serum alpha-L-fucosidase: a new marker for the diagnosis of primary hepatic cancer? *Hepatology*, **4**, 889.

DICIOCCIO, R.A., BARLOW, J.J. & MOTTA, K.L. (1985). Evaluation of alpha-L-fucosidase as a marker of primary liver cancer. *IRCS Med. Sci.*, **13**, 849.

DURAND, P., BORRONE, C. & DELTA CELLA, G. (1969). Fucosidosis. *J. Pediatr.*, **75**, 665.

GUILLOU, H., DAVID, V., LORY, Y., LETRENT, A., ALLANIC, H. & LEGALL, J. (1982). Serum lysosomal acid hydrolase activities in Grave's disease. *Clin. Chim. Acta*, **120**, 219.

HERMAN, A.A.B., IRWIG, L.M. & GROENEWALD, H.T. (1988). Evaluating obstetric risk scores by receiver operating characteristic curves. *Am. J. Epidemiol.*, **127**, 831.

HOLMES, E.H. & HAKOMORI, S.I. (1982). Isolation and characterisation of a new fucoganglioside accumulated in precancerous rat liver and in rat hepatoma induced by *N*-2-acetylaminofluorine. *J. Biol. Chem.*, **257**, 7698.

KEW, M.C. & NEWBERNE, P.M. (1982). Tumor markers in man. In *Hepatocellular Carcinoma, UICC Technical Report Series, Vol. 74*, Okuda, K. & Mackay, I.R. (eds) p. 127. UICC: Geneva.

LYNCH, H.T., SHUELKE, G.S., WELLS, I.C. & 5 others (1985). Hereditary ovarian carcinoma. *Cancer*, **55**, 410.

REGLERO, A., CARRETERO, M.I. & CABEZAS, J.A. (1980). Increased serum alpha-L-fucosidase and $\beta$-*N*-acetylglucosimidase activities in diabetic, cirrhotic and gastric cancer patients. *Clin. Chim. Acta*, **103**, 155.

ROBINSON, D. & THORPE, R. (1973). Human liver alpha-L-fucosidase. *Clin. Chim. Acta*, **47**, 403.

RUPERT, G. & MILLER, J. (1981). *Simultaneous Statistical Inference*, 2nd edn, p. 67. Springer-Verlag: Berlin.

TROOST, J., VAN DER HEIJDEN, M.C.M. & STAAL, G.E.J. (1976). Characterisation of alpha-L-fucosidase from two different families with fucosidosis. *Clin. Chim. Acta*, **73**, 329.

TURNER, B.U., TURNER, V.S., BERATIS, N.G. & HIRSCHHORN, K. (1975). Polymorphism of human alpha-L-fucosidase. *Am. J. Hum. Genet.*, **27**, 651.

VISCHER, P. & REUTTER, W. 1978). Specific patterns of fucoprotein biosynthesis in the plasma membranes of Morris hepatoma 7777. *Eur. J. Biochem.*, **84**, 363.